# *Thymine DNA Glycosylase (TDG)* is involved in the pathogenesis of intestinal tumors with reduced *APC* expression

**DOI:** 10.18632/oncotarget.21219

**Published:** 2017-09-23

**Authors:** Jinfei Xu, Salvatore Cortellino, Rossella Tricarico, Wen-Chi Chang, Gabrielle Scher, Karthik Devarajan, Michael Slifker, Robert Moore, Maria Rosaria Bassi, Elena Caretti, Margie Clapper, Harry Cooper, Alfonso Bellacosa

**Affiliations:** ^1^ Cancer Epigenetics Program, Fox Chase Cancer Center, Philadelphia, PA 19111, USA; ^2^ Cancer Prevention and Control Program, Fox Chase Cancer Center, Philadelphia, PA 19111, USA; ^3^ Department of Biostatistics, Fox Chase Cancer Center, Philadelphia, PA 19111, USA; ^4^ Department of Pathology, Fox Chase Cancer Center, Philadelphia, PA 19111, USA

**Keywords:** TDG, APC, colorectal cancer, intestinal cancer

## Abstract

Thymine DNA Glycosylase (TDG) is a base excision repair enzyme that acts as a thymine and uracil DNA N-glycosylase on G:T and G:U mismatches, thus protecting CpG sites in the genome from mutagenesis by deamination. In addition, TDG has an epigenomic function by removing the novel cytosine derivatives 5-formylcytosine and 5-carboxylcytosine (5caC) generated by Ten-Eleven Translocation (TET) enzymes during active DNA demethylation. We and others previously reported that *TDG* is essential for mammalian development. However, its involvement in tumor formation is unknown. To study the role of *TDG* in tumorigenesis, we analyzed the effects of its inactivation in a well-characterized model of tumor predisposition, the *Apc*^Min^ mouse strain. Mice bearing a conditional *Tdg*^flox^ allele were crossed with *Fabpl*::Cre transgenic mice, in the context of the *Apc*^Min^ mutation, in order to inactivate *Tdg* in the small intestinal and colonic epithelium. We observed an approximately 2-fold increase in the number of small intestinal adenomas in the test *Tdg*-mutant *Apc*^Min^ mice in comparison to control genotypes (p=0.0001). This increase occurred in female mice, and is similar to the known increase in intestinal adenoma formation due to oophorectomy. In the human colorectal cancer (CRC) TCGA database, the subset of patients with *TDG* and *APC* expression in the lowest quartile exhibits an excess of female cases. We conclude that *TDG* inactivation plays a role in intestinal tumorigenesis initiated by mutation/underexpression of *APC*. Our results also indicate that *TDG* may be involved in sex-specific protection from CRC.

## INTRODUCTION

Cytosine and 5-methylcytosine (5mC) are intrinsically unstable in genomic DNA and have a tendency to spontaneously deaminate, generating thymine and uracil, respectively, which, if not removed from the G:T and G:U mismatches prior to replication, will lead to incorporation of adenine [1, 2]. 5mC is mostly located at palindromic CpG sequences, and this mechanism of CpG site mutagenesis by deamination is estimated to cause nearly one-third of all mutations in both cancer and human genetic diseases [3, 4]. In particular, through recent next-generation sequencing, transition mutations at NpCpG sites are being recognized as the most frequent mutational signature across the vast majority of cancer types [5]. In order to maintain genomic stability at CpG sites, two base excision repair enzymes, Thymine DNA Glycosylase (TDG) and Methyl-Binding Domain 4 (MBD4), remove the offending thymine and uracil with their DNA *N-*glycosylase activity [6–9].

In addition to its role in genomic stability of CpG sites, TDG is also required for active DNA demethylation during development; TDG balances the activity of DNA methyltransferases by maintaining CpG islands in their unmethylated state and promoting demethylation of tissue-specific, developmentally regulated enhancers [10, 11]. In this role in epigenomic stability, TDG acts downstream the Ten-Eleven Translocation (TET) family of dioxygenases in pathways of DNA demethylation initiated by iterative oxidation of 5mC to the novel cytosine species: 5-hydroxymethylcytosine (5hmC), 5-formylcytosine (5fC) and 5-carboxylxytosine (5caC). In fact, TDG is the main biochemical activity involved in removal of 5fC and 5caC [9, 12]. It should be noted that a similar role of MBD4 in DNA demethylation has been proposed but remains controversial; in fact, whereas TDG is essential [10, 13], MBD4 is dispensable for mammalian development [9].

Mutagenesis by deamination is thought to play an important role in human colorectal cancer (CRC); CRC has a high frequency of mutations at NpCpG sequences (so-called signature 1 mutations), and half of *TP53* inactivating mutations, which are critical alterations for CRC progression [14], are G:C to A:T transitions within a CpG site [5, 15–17]. In addition, altered methylation patterns (epimutations) are known to be involved in the pathogenesis of CRC with genome-wide hypomethylation and hypermethylation and silencing of CpG-rich sequences (CpG islands) at promoters of tumor suppressor and other genes [18, 19]. Thus, MBD4 and/or TDG defects may contribute to CRC formation.

Indeed, we and others have shown that *MBD4* is frequently mutated in microsatellite unstable, mismatch repair (MMR)-defective CRC [20–22]; and affects the mutational landscape independent of the MMR defect [23]. Moreover, enhanced tumorigenicity due to MBD4 inactivation could be demonstrated by breeding *Mbd4* mutant mice with the *Apc*^Min^ mouse [24, 25], which is a very sensitive reporter system to score genetic interactions with the *Apc* tumor suppressor gene [26, 27]. However, despite its presumed role in genomic and epigenomic instability, the involvement of *TDG* in cancer, and in particular in CRC, is poorly characterized, with the exception of a study in which *TDG* inactivation in a rectal carcinoma from a patient with constitutional MMR deficiency increased the number of C>T transitions at CpG sites [28]. In this article, we conducted human and mouse studies, which concordantly establish a role for inactivation of *TDG*, as an important tumor suppressor in the pathogenesis of a subset of intestinal tumors.

## RESULTS

### Generation of mice bearing a conditional *Tdg* knock-out allele and intestinal inactivation of *Tdg* in the context of the *Apc*^Min^ mutation

To study the role of TDG in intestinal tumor formation, we conditionally inactivated *Tdg* in the small intestine and colon. Tissue-specific conditional inactivation was necessary in order to bypass embryonic lethality associated with *Tdg* inactivation in the germline [10, 13]. We previously described the generation of mice bearing the inactive, null allele *Tdg*^-^, in which Cre-mediated recombination between the *loxP* sites generates a deletion of exons 3 through 7 [10]. Mice with the conditional *Tdg* allele (*Tdg*^flox^), were generated by crossing mice bearing the original recombined locus (*Tdg*^neoflox^) with mice expressing the enhanced Flp recombinase at the Rosa26 locus (Rosa26::FlpeR) [29]. Through this cross, Flp-mediated recombination of the *frt* sites excises the *neo* gene, generating a *Tdg*^flox^ allele with *loxP* sites flanking exons 3 and 7 (Supplementary Figure 1). Mice homozygous for the *Tdg*^flox^ allele do not exhibit lethality or infertility, and age normally (data not shown), thus confirming that this allele is indeed conditional.

Finally, mice bearing the conditional *Tdg*^flox^ allele were crossed with *Fabpl*::Cre transgenic mice, which yields mosaic gene inactivation in the small intestine and colon [30, 31], and with the Fox Chase Cancer Center strain of *Apc*^Min^ mice, *Apc*^Min-FCCC^ [32]. All the mice were maintained on a nearly pure C57/BL6 background (see Material and Methods for details).

### Increased adenoma formation in *Fabpl::*Cre^+/o^ *Tdg*^flox/-^ *Apc*^Min/+^ mice

**Figure 1:**
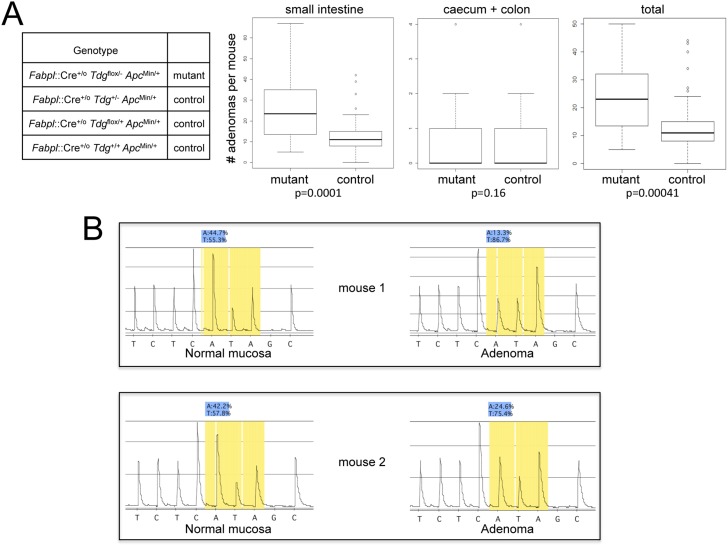
Increased intestinal tumor formation in Tdg conditional knock-out mice crossed into the *Apc*^Min^ background **(A)** Box plot representation of the distributional characteristics of the number of small intestinal, caecum plus colonic, and total adenomas in experimental *Fabpl*::Cre^+/o^ *Tdg*^flox/-^ *Apc*^Min/+^ (mutant) and *Fabpl::*Cre^+/o^ *Tdg^+/-^* *Apc*^Min/+^, *Fabpl::*Cre^+/o^ *Tdg*^+/flox^ *Apc*^Min/+^ and *Fabpl::*Cre^+/o^ *Tdg*^+/+^ *Apc*^Min/+^ (control) mice; the line in the box represents the median. The *p* value was determined by two-sided Mann-Whitney test. The average tumor numbers and standard deviations are as follows: small intestinal, mutant: mean=26, SD=15.68; small intestinal, control: mean=12.76, SD=8.95; colon, mutant: mean=0.84, SD=1.26; colon, control: mean=0.41, SD=0.74; total, mutant: mean=23.84, SD=12.70; total, control: mean=13.16, SD=9.35. **(B)** Pyrogram of the complementary sequence around the *Apc*^Min^ mutation showing evidence of loss of heterozygosity in two adenomas from *Fabpl*::Cre^+/o^ *Tdg*^flox/-^ *Apc*^Min/+^ mice in comparison to normal mucosa. The *Apc*^Min^ mutation, a T→A point mutation at base 2,549, is present in heterozygosity (approximately 50%) in normal mucosa of *Fabpl*::Cre^+/o^ *Tdg*^flox/-^ *Apc*^Min/+^ mice, and increases to approximately 75-85% in adenomas.

A total of 67 mice were divided into four groups; the test cohort with genotype *Fabpl::*Cre^+/o^ *Tdg*^flox/-^ *Apc*^Min/+^, and the control cohorts with genotypes *Fabpl::*Cre^+/o^ *Tdg^+/-^* *Apc*^Min/+^, *Fabpl::*Cre^+/o^ *Tdg*^+/flox^ *Apc*^Min/+^ and *Fabpl::*Cre^+/o^ *Tdg*^+/+^ *Apc*^Min/+^. Mice were aged and sacrificed at 150 days, as done in previous studies of the Fox Chase Cancer Center strain of the *Apc*^Min^ mouse [32], and the total number of gross and microscopic adenomas was counted. The results revealed that the test *Tdg*^flox/-^ mice exhibit an approximately two-fold increase in the number of small intestinal adenomas in comparison to control genotypes (p=0.0001) (Figure 1A). No difference was seen in the number of caecum and colonic adenomas (Figure 1A). No carcinomas were seen.

To evaluate whether *Tdg* deletion alters the mechanism of inactivation of the second (normal) copy of the *Apc* gene in adenomas, a total of 4 matched normal colonic mucosa and adenoma samples from *Fabpl*::Cre^+/o^ *Tdg*^flox/-^ *Apc*^Min/+^ mice were analyzed for *Apc* loss of heterozygosity by pyrosequencing around the *Apc*^Min^ mutation. We found that all the adenomas tested exhibited loss of heterozygosity (Figure 1B), as in the classical model of intestinal tumorigenesis in *Apc*^Min/+^ mice [33].

**Figure 2:**
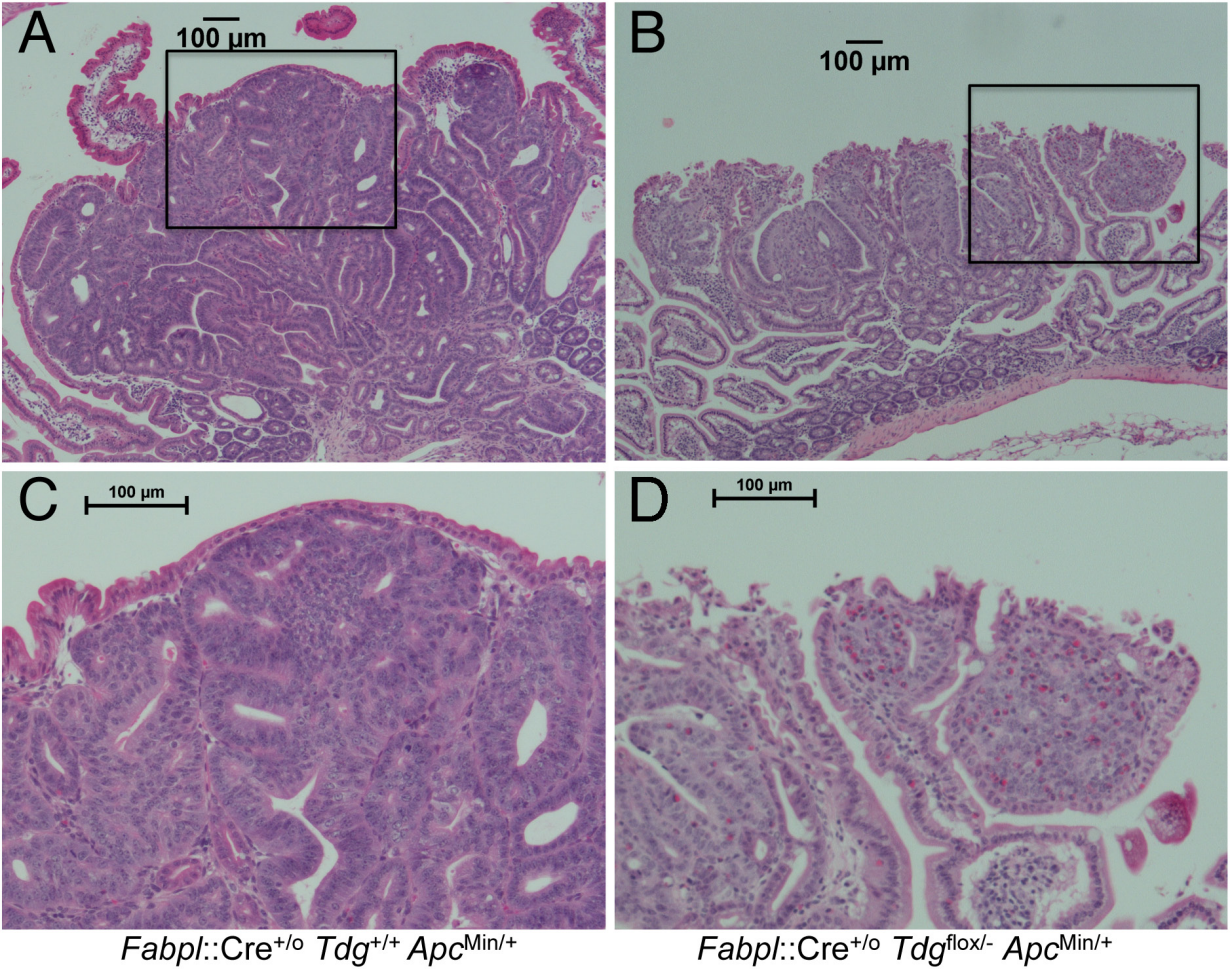
Histopathology of poorly differentiated small intestinal adenomas in *Fabpl*::Cre^+/o^ *Tdg*^flox/-^ *Apc*^Min/+^ mice Representative examples of morphological differences between typical well-differentiated adenomas in *Fabpl*::Cre^+/o^ *Tdg*^+/+^ *Apc*^Min/+^ (*Tdg* wild type) **(A)** and poorly differentiated adenomas in *Fabpl*::Cre^+/o^ *Tdg*^flox/-^ *Apc*^Min/+^ mice (*Tdg* conditional knock-out) **(B)**. The respective insets **(C** and **D)** show an enlarged area. Poorly differentiated adenomas in *Tdg* conditional knock-out mice manifest vesicular nuclei with prominent nucleoli and infiltration of eosinophils.

Pathological examination of small intestinal adenomas from the test *Fabpl*::Cre^+/o^ *Tdg*^flox/-^ *Apc*^Min/+^ mice in comparison to adenomas from control *Fabpl*::Cre^+/o^ *Tdg*^+/+^ *Apc*^Min/+^ mice revealed an excess of poorly differentiated tumors, in which the differentiated gland (crypt/villus) architecture had been lost (Figure 2). Specifically, 16/44 (36.4%) adenomas examined displayed undifferentiated features in *Fabpl*::Cre^+/o^ *Tdg*^flox/-^ *Apc*^Min/+^ mice *vs*. 8/74 (10.8%) in *Fabpl*::Cre^+/o^ *Tdg*^+/+^ *Apc*^Min/+^ mice (p=0.0017, Fisher's exact test). In addition, adenomas in *Tdg*-mutant mice were characterized by vesicular nuclei with prominent nucleoli and infiltration of eosinophils (Figure 2B, 2D). This histology indicates a possible role for TDG in the establishment or maintenance of the differentiated state of the small intestine.

### The increased adenoma number in *Fabpl::*Cre^+/o^ *Tdg*^flox/-^ *Apc*^Min/+^ mice is linked to hormonal effects

When we divided the test and control mice by sex, we noticed that the increased adenoma formation associated with the *Fabpl::*Cre^+/o^ *Tdg*^flox/-^ *Apc*^Min/+^ genotype was more prevalent in female mice (Table 1), indicating an involvement of sex hormones in the mechanism of action of *Tdg* inactivation. Since TDG is a known co-activator of estrogen receptor (ER) α [34] and β [35], and ovariectomy increases adenoma formation in *Apc*^Min^ mice [36], it is possible that the known protective effect of female hormones, and especially estrogens, on CRC formation [37] is TDG-dependent. To test this possibility, female mice of the test *Fabpl::*Cre^+/o^ *Tdg*^flox/-^ *Apc*^Min/+^ genotype and the three control genotypes underwent ovariectomy at 5-6 weeks and were scored for intestinal adenoma formation at 150 days (Table 2). Importantly, a similar increased number of adenomas (in comparison to the average number of adenomas in control mice not undergoing ovariectomy, Table 1) was detected in both the test *Fabpl::*Cre^+/o^ *Tdg*^flox/-^ *Apc*^Min/+^ genotype group and the control genotype groups (Table 2), indicating that ovariectomy is, in a way, “epistatic” with the mutant *Tdg* genotype, i.e. the protective effect of female hormones on intestinal tumor formation may be largely TDG-dependent.

**Table 1:** Sex-dependent impact of *Tdg* conditional mutation on adenoma formation

Genotype	Female mice sacrificed	Total no. of tumors	Average no. of tumors per mouse	Male mice sacrificed	Total no. of tumors	Average no. of tumors per mouse
*Fabpl*::Cre^+/o^ *Tdg*^flox/-^ *Apc*^Min/+^	11	344	31.3	8	123	15.4
*Fabpl*::Cre^+/o^ *Tdg*^+/-^ *Apc*^Min/+^	8	96	12	10	107	10.7
*Fabpl*::Cre^+/o^ *Tdg*^flox/+^ *Apc*^Min/+^	9	124	13. 8	14	163	11.6
*Fabpl*::Cre^+/o^ *Tdg*^+/+^ *Apc*^Min/+^	7	90	12.9	9	105	11. 7

**Table 2:** Effect of ovariectomy on intestinal adenoma formation in *Tdg* mutant and control mice

Genotype	Female mice sacrificed	Total no. of tumors	Average no. of tumors per mouse	Average no. of tumors per female mouse *(from Table 1)*
*Fabpl*::Cre^+/o^ *Tdg*^flox/-^ *Apc*^Min/+^	13	359	27.6	31.3
Control mice (*combined:*	7	166	23.7	12.9
*Fabpl*::Cre^+/o^ *Tdg*^+/-^ *Apc*^Min/+^				
*Fabpl*::Cre^+/o^ *Tdg*^flox/+^ *Apc*^Min/+^				
*Fabpl*::Cre^+/o^ *Tdg*^+/+^ *Apc*^Min/+^)				

### Involvement of *TDG* in human colorectal cancer

To evaluate the role of *TDG* in human CRC, we first examined the expression of TDG in CRC cell lines of the NCI-60 panel. Western blot analysis revealed that the levels of TDG varied from high to low in these cell lines (Supplementary Figure 2), indicating a complex role in CRC formation.

**Figure 3:**
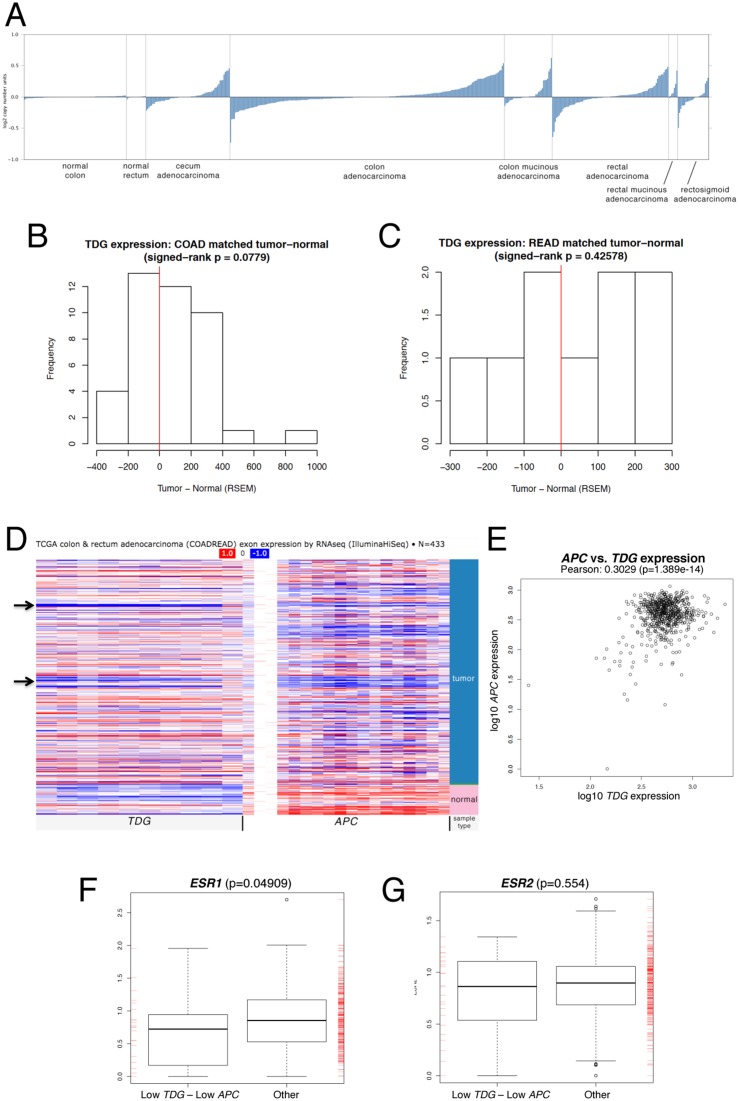
Relevance of *TDG* expression to human CRC **(A)** Expression levels of *TDG* mRNA in normal colon and rectum, and corresponding adenocarcinomas (indicated) of the TCGA COAD (colon adenocarcinoma) and READ (rectal adenocarcinoma) data sets (Oncomine). Expression levels of *TDG* mRNA in matched normal-tumor samples of the TCGA COAD (colon adenocarcinoma) **(B)** and READ (rectal adenocarcinoma) **(C)** data sets. **(D)** Heatmap (UCSC Cancer Browser) showing expression of *TDG* and *APC* mRNA in normal and tumor samples from the TCGA COADREAD data sets. Arrows indicate group of tumors with reduced expression of both *TDG* and *APC*. **(E)** Pearson correlation of *TDG* and *APC* mRNA expression levels in tumor samples from the TCGA COADREAD data sets. Box plot representation of the *ESR1* **(F)** and *ESR2* **(G)** mRNA expression levels in TCGA COADREAD samples with low quartile of both *TDG* and *APC* expression *vs*. other samples. The *p*-value was determined by two-sided Mann-Whitney test. A significant difference was found for *ESR1* but not *ESR2*.

Examination of the TCGA COAD (colon adenocarcinoma) and READ (rectal adenocarcinoma) data sets also revealed a wide range of *TDG* mRNA expression ratios between normal and tumor pairs in matched samples (Figure 3A-3C). Interestingly, we noticed a trend towards a correlation between low *TDG* and low *APC* expression, which validates the relevance of our mouse model (Figure 3D, 3E).

Importantly, in 618 sporadic CRC samples processed on both RNA-Seq platforms (HiSeq & GenomeAnalyzer), the cases with lowest quartile of expression for both *TDG* and *APC* comprised a subset that is overrepresented by women *vs*. men (Table 3, p = 1.897e-05), further validating the mouse model. Such overrepresentation was detected even when the analysis was limited to 372 CRC samples processed on the HiSeq platform only (Table 3, p = 0.03333). In keeping with the potential role of TDG in mediating the protective effects of estrogen, analysis of the TCGA data revealed a correlation between the lowest quartile of *TDG* and *APC* expression, and low expression of the ERα gene (*ESR1*) (Figure 3F); the correlation with the ERβ gene (*ESR2*) was not significant (Figure 3G).

**Table 3:** Sex distribution of TCGA COADREAD tumors based on low quartile of *TDG* and *APC* expression

	HiSeq & GenomeAnalyzer		HiSeq only	
Group	Female	Male	Female	Male
Low *TDG* - Low *APC*	39	13	16	8
Other	250	316	151	197
*p* value	1.897e-05		0.03333	

In TCGA data, overall survival was not linked to the levels of *TDG* expression (Supplementary Figure 3A), although the subset of patients with the high quartile of *TDG* expression exhibited a trend towards increased overall survival in comparison to patients of the remaining three quartiles (Supplementary Figure 3B). Also, the subset of patients with *TDG* and *APC* expression in the lowest quartile does not show different survival from all the other patients (Supplementary Figure 3C).

**Figure 4:**
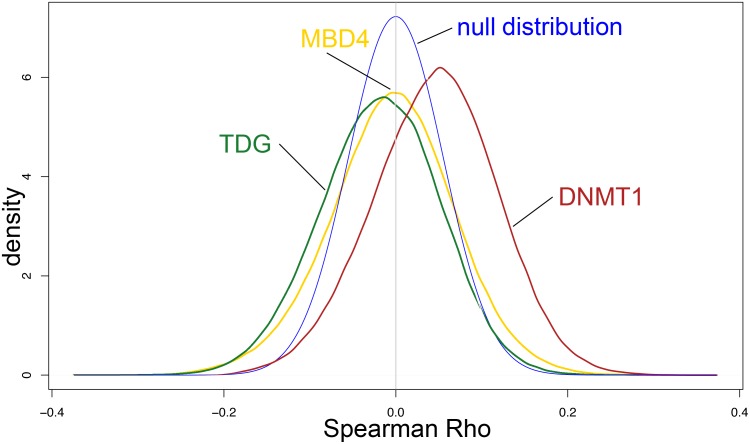
Impact of *TDG*, *MBD4* and *DNMT1* expression on epigenome of CRC cases from the TCGA database Spearman correlations of *TDG*, *MBD4* and *DNMT1* expression with Infinium 450K probe beta values. The null distribution is indicated. Methylation levels at more sites are negatively correlated with *TDG* expression than are positively correlated, thus causing a shift of the plot to the left of the null distribution curve. Conversely, *DNMT1* expression correlates positively with methylation levels, thus causing a shift of the plot to the right of the null distribution curve. *MBD4* expression did not show any preferential correlation.

TDG has a prominent role in epigenomic regulation by acting downstream of the TET enzymes in DNA demethylation pathways [9]. Therefore, we wished to determine whether altered expression of *TDG* relates to changes in DNA methylation in CRC. We mined the TCGA CRC DNA methylation data and correlated the expression levels of *TDG* and, as controls, *DNMT1* and *MBD4*, to the beta values (methylation levels) for all the Infinium 450K CpG sites. The results revealed a distribution of correlations in which methylation levels at more sites are negatively correlated with *TDG* expression than are positively correlated, as expected for the role of TDG in DNA demethylation (Figure 4). Conversely, *DNMT1*, acting as a positive control for this analysis, correlated mostly positively with methylation levels, in keeping with its enzymatic activity; *MBD4* did not show any preferential correlation (Figure 4). These results indicate that altered expression of *TDG* has an impact on the degree of DNA methylation of CRC.

## DISCUSSION

The combined results from our *in vivo* mouse studies and investigation of human cancer databases indicate a role of *TDG* in the suppression of intestinal tumorigenesis, and particularly in sex-specific protection from CRC. In order to study the role of *TDG* in tumor formation, we generated mice bearing the conditional *Tdg*^flox^ allele to bypass embryonic lethality due to germ line inactivation, and took advantage of *Fabpl*::Cre transgenic mice for gene inactivation in the mouse intestine. To score unequivocally for a role of *TDG* in intestinal tumor formation, we used the reliable model represented by the *Apc*^Min^ mice, which demonstrated a two-fold increase in the number of small intestinal adenomas in *Tdg* conditionally mutant mice in comparison to control mice.

Western blot analysis of a series of human CRC cell lines revealed a wide range of TDG expression levels. However, it has been reported that the expression levels of TDG are maintained, or even increased, in CRC samples in comparisons to normal colonic mucosa [38]. To resolve this apparent paradox, we analyzed the TCGA expression data and found that while the overall levels of *TDG* are maintained or increased in CRC, there exists a subset of cases characterized by reduced expression. Remarkably, this subset is also characterized by reduced *APC* expression, which lends functional significance to our model of mutant *Tdg* x *Apc*^Min^ mice.

An important uniformity of our mouse model and the human CRC subset is the sex bias. Both mutant *Tdg* x *Apc*^Min^ mice and the low *TDG*/low *APC* patient subset are characterized by an excess of female cases, which indicates that TDG may normally mediate the protective effects of estrogen on intestinal tumor formation. ERα and ERβ are known modifiers of APC-dependent tumorigenesis, and their inactivation increases intestinal tumor formation in the *Apc*^Min^ mice [36, 39, 40]. Since TDG is a co-activator of ERα [34] and ERβ [35], it is possible that TDG participates to the protective effects of estrogen by fully promoting ERα- and ERβ-mediated transcriptional activation.

Interestingly, adenomas in *Tdg* mutant mice show an excess of undifferentiated features in comparison to control mice, and a characteristic infiltration of eosinophils, which points to a possible role of *Tdg* in maintaining the proper intestinal architecture and avoiding abnormal chemotactic stimuli. In addition, the solid, undifferentiated architecture and nuclear/nucleolar morphology is reminiscent of the histology of human CRC with the so-called CpG island methylator phenotype (CIMP) (37, 102), which raises the possibility that loss of TDG demethylating activities may promote the onset of tumors characterized by altered methylation. A potential confirmation of this contention is the fact that in the TCGA CRC dataset there exists an inverse correlation between *TDG* expression and DNA methylation levels, which indicates that the role of TDG in intestinal tumor formation may be linked to its function in DNA demethylation, i.e. to maintenance of epigenomic stability.

On the other hand, TDG DNA repair activity may also play a role in intestinal tumor formation, via maintenance of genomic stability of CpG sites, i.e. the subset of tumors with low TDG expression may exhibit high levels of transition mutations at CpG sites. However, the fact that the second allele of *Apc* is inactivated by loss of heterozygosity rather than by point mutations, as is the case for *Mbd4* mutant mice [24, 25], argues against this possibility. Be as it may, additional studies are warranted that will more directly examine the mutational impact of reduced TDG activity.

It has been suggested that TDG is a positive regulator of WNT/β-catenin signaling in CRC [38, 41]. On the other hand, TCGA mutational profiling of CRC revealed the presence of mutations in several WNT pathway genes, often in combination with *APC* mutations, indicating that multiple alterations of WNT signaling are needed for CRC formation and may be under positive selection [42]. Thus, the CRC subset with low *TDG* expression levels may impair WNT signaling and promote tumorigenesis in combination with *APC* mutation/reduced expression.

In summary, these observations support a role of reduced *TDG* expression in the pathogenesis of CRC. However, it remains to be determined the significance of *TDG* in those CRC cases that manifest maintained/increased *TDG* expression; these cases may underscore the need for tumor cells to retain or enhance a hitherto unclear function of this gene that in fact may potentially represent a novel vulnerability of cancer.

## MATERIALS AND METHODS

### Mice, pathological analysis and histopathology

Mice bearing the null *Tdg* allele (*Tdg*^-^) and mice harboring the original *Tdg* recombined locus, that includes the neo^R^ gene and the *loxP* and *frt* recombination sites (*Tdg*^neoflox^), have been previously described (10). Mice expressing the enhanced Flp recombinase at the Rosa26 locus (Rosa26::FlpeR) [29], *Fabpl*::Cre transgenic mice [30, 31], and the Fox Chase Cancer Center strain of the *Apc*^Min^ mice, *Apc*^Min-FCCC^ [32] have also been described and maintained on a C57/BL6 background.

Initially, mice bearing the conditional *Tdg* allele (*Tdg*^flox^), generated by crossing *Tdg*^neoflox^ mice with Rosa26::FlpeR and *Tdg*^-^ mice, had a mixed genetic background that was approximately 75% C57/BL6 and 25% 129/Sv-J. Subsequent experiments were conducted after at least 10 backcrosses into C57/BL6, therefore the genetic background of these two strains was approximately 99.8% C57/BL6 and 0.2% 129/Sv-J.

Mice were housed in the Fox Chase Cancer Center Laboratory Animal Facility, a fully accredited facility, and all experiments were approved by the Fox Chase Cancer Center Institutional Animal Care and Use Committee.

All the genotyping was conducted by PCR analysis of mouse tail genomic DNA; primer sequences and PCR conditions are available upon request.

Mice were euthanized by CO_2_ asphyxiation and examined at 150 days. For pathological examination, the small and large intestine were excised, opened with a longitudinal cut and rinsed in phosphate buffer saline. Small and large intestine were examined under a dissecting microscope and gross lesions were counted. For histopathology, the small intestine was analyzed by the “jelly roll” method, a procedure in which the tissue is rolled on a stick before fixation in buffered formalin, paraffin-embedding, sectioning and hematoxylin/eosin staining; whereas the cecum and colorectum were cut in cross sections, in a “bread loaf” fashion, at 2mm intervals. Adenomas were verified microscopically using established criteria [43].

### Ovariectomy

Sexually immature female mice (5-6 week old) were anesthetized with isoflurane and ovariectomized bilaterally. A skin incision was made on the dorsal surface of mice to expose and remove the ovaries. After the operation, the mice were kept on a warm platform for 2 hours until their complete recovery.

### Analysis of loss of heterozygosity of *Apc* by pyrosequencing

A region of the mouse Apc gene around the Min mutation was amplified by polymerase chain reaction using the following primers 5’Bio-CCT CAA GGG GAA GTT TAG ACA GTT-3’ and 5’- GAT GGT AAG CAC TGA GGC CAA TA -3’. The 5’-biotinylated forward primer for the pyrosequencing reaction was isolated using streptavidin-coated Sepharose beads (Amersham Biosciences, Piscataway, NJ) and the PSQ 96 Sample Preparation kit (Biotage,Westborough, MA). The single-strand DNA template was incubated with sequencing primer 5’- CTG AGG CCA AT ACCT CG -3’. The sequencing by synthesis reaction of the complementary strand was performed on a PSQ 96MA instrument (Biotage) using PyroGold reagents (Biotage).

### Cell culture and western blot analysis

The CRC cell lines of the NCI-60 panel were grown in RPMI supplemented with 10% fetal bovine serum. Western blotting was conducted as previously described (10). Briefly, cells were lysed in RIPA buffer (50 mM Tris HCl pH 7.4, 150 mM NaCl, 1% sodium deoxycholate, 1% Triton X-100, 0.1% SDS, 10 mM NaF, 1 mM each of sodium pyrophosphate, sodium orthovanadate, dithiothreitol, and EDTA), plus protease inhibitors. Lysates were fractionated by SDS-PAGE and transferred to PVDF membranes (Millipore). Membranes were blocked in 4% nonfat dry milk in PBS and incubated with anti-Tdg antibody (gift of Dr. K. Sugasawa), 1/400 dilution, or anti-β-actin (Abcam), 1/10,000 dilution, in 2% nonfat dry milk in PBS. Detection was performed using enhanced chemiluminescence (Amersham).

### Statistical analysis

The two-sided Mann-Whitney test was used to compare the number of adenomas between mutant and control groups. For the box-plots shown in Figures 1B, 3F, and 3G, the height of the box represents the inter-quartile range (IQR), where the upper and lower ends indicate the third and first quartiles, respectively. The solid black horizontal line inside the box represents the median value while the whiskers (the two solid horizontal lines at either end, connected by dotted lines) extend to the most extreme data points which are no more than 1.5 times the IQR from the box in each direction. Using this criterion, the points that lie beyond these whiskers are considered to be outliers.

The Wilcoxon signed-rank test was used to compare gene expression intensities between matched tumor and normal samples, Comparison of overall survival between groups representing levels of TDG expression was performed via Kaplan-Meier analysis; the log-rank test was used to assess significance. Fisher's exact test was used to test association between gender and membership in the low *TDG*/low *APC* group.

A Type I Error of 5% was used to determine statistical significance.

### Bioinformatics analysis

Level 3 clinical, RNA-seq, and Illumina 450K methylation data for colon adenocarcinoma (COAD) and rectum adenocarcinoma (READ) were downloaded from the TCGA data portal in January, 2016. RSEM gene-level normalized counts and beta-values (ratio of methylated probe intensity to overall intensity for each interrogated site) were used as gene expression values and methylation measurements, respectively.

## SUPPLEMENTARY MATERIALS FIGURES


