# Supplementary material for: Thymine DNA Glycosylase (TDG) is involved in the pathogenesis of intestinal tumors with reduced APC expression
Source: Oncotarget. 2017 Sep 23;8(52):89988–97. doi: 10.18632/oncotarget.21219 (PMC5685726; doi:10.18632/oncotarget.21219)
Supplement: Supplementary file 1 [file oncotarget-08-89988-s001.pdf]

Thymine DNA Glycosylase (TDG) is involved in the pathogenesis of intestinal tumors with reduced *APC* expression

SUPPLEMENTARY MATERIALS

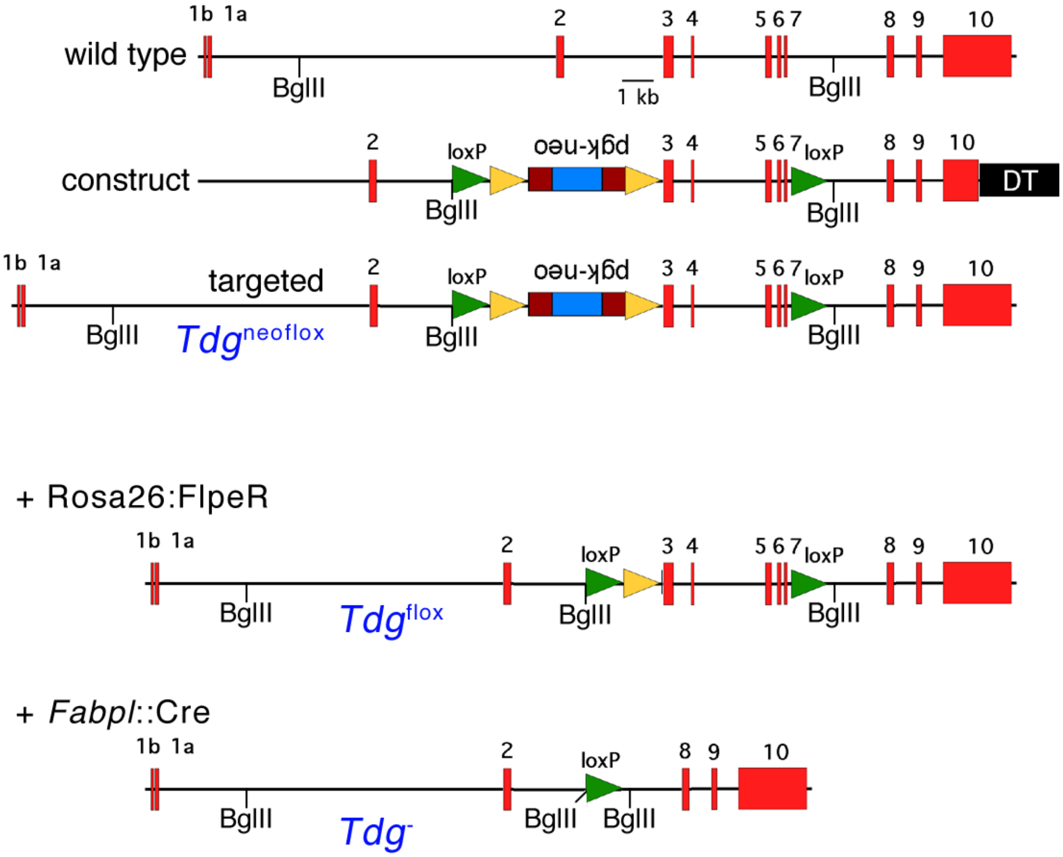

Supplementary Figure 1: Production of the conditional *Tdg*^{flox} allele. In the *Tdg* targeting construct, a pgk-neo cassette flanked by *FRT* sites (yellow triangles) along with a *loxP* site (green triangle) is located between exons 2 and 3, while a *loxP* site is placed immediately downstream of exon 7; the diphteria toxin gene is marked as DT. The targeted recombinant *Tdg*^{neoflox} allele was produced in ES cells after homologous recombination and allowed the generation of heterozygous *Tdg*$^{neoflox/+}$ mice. The conditional *Tdg*^{flox} allele, in which Flp-mediated recombination between the *FRT* sites removes the neo^{R} cassette, was produced by crossing *Tdg*$^{neoflox/+}$ mice with Rosa26::FlpeR transgenic mice. By crossing with *Fabpl*::Cre transgenic mice, the *Tdg*^{flox} allele is converted in the null *Tdg*$^{-}$ allele (deletions of exons 3 through 7).

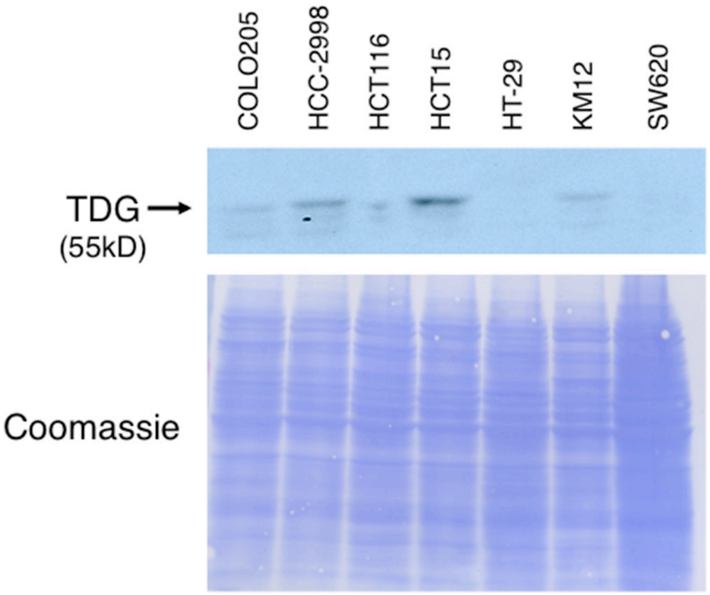

Supplementary Figure 2: Levels of TDG protein in CRC cell lines of the NCI-60 panel. Detection of TDG protein by western blotting (top panel) with an antibody against TDG. A Coomassie-stained gel (bottom panel) reveals that approximately equal amounts of protein lysate were loaded in each lane, except the last lane (SW620); despite the higher amounts of SW620 lysate loaded, the levels of TDG are very low in this cell line.

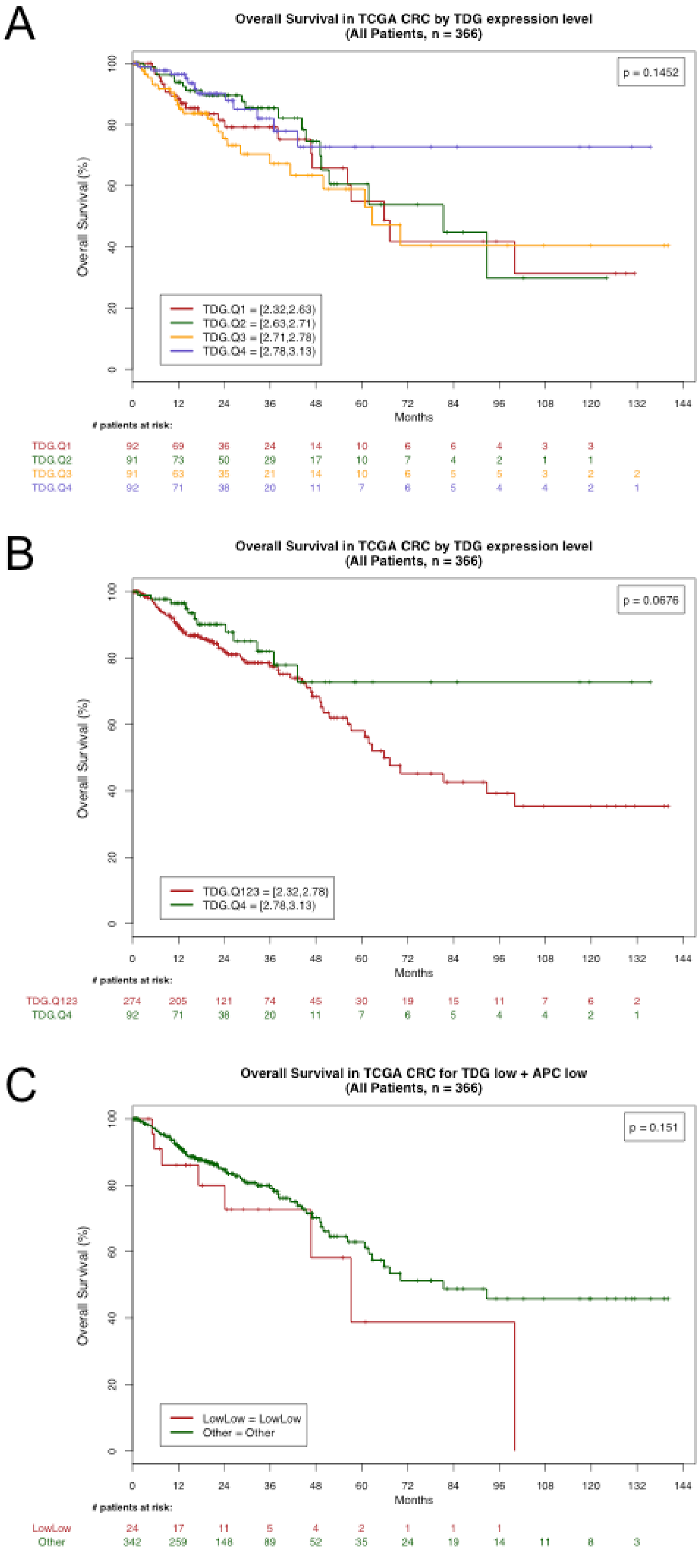

Supplementary Figure 3: Survival analysis of patients in the TCGA COADREAD dataset according to *TDG* mRNA expression levels. **(A)** Kaplan-Meier survival analysis according to the quartile of *TDG* mRNA expression; comparison of survival of the subset of patients within each quartile of TDG expression. **(B)** Kaplan-Meier survival analysis according to the quartile of *TDG* mRNA expression; comparison of survival of the subset of patients with high quartile of TDG expression *vs.* other quartiles combined together. **(C)** Kaplan-Meier survival analysis of the subset of patients with low quartile of both *TDG* and *APC* expression *vs.* other samples.
